# Clinical, electrophysiological, and prognostic study of postinjection sciatic nerve injury: An avoidable cause of loss of limb in the peripheral medical service

**DOI:** 10.4103/0972-2327.53081

**Published:** 2009

**Authors:** Wani Maqbool, Saleem Sheikh, Asrar Ahmed

**Affiliations:** Department of Neurology, S. K. Institute of Medical Sciences, Soura, J & K, India

**Keywords:** Sciatic nerve, neuropathy, post-injection injury

## Abstract

**Background:**

Post injection sciatic nerve injury is a common cause of sciatic nerve mononeuropathy in the developing world largely due to inadequate health care facilites in the rural regions.

**Objective:**

The study was conducted to analyse the pattern of this nerve lesion in clinical and electrophysiological parameters and also to study the outcome in a conservatively treated cohort.

**Materials and Methods:**

One hundred and six patients who underwent evaluation at our laboratory from 2000 to 2006 for post injection sciatic neuropathy formed the study population. Twenty two of these were followed up (mean 6.6 months) for the outcome.

**Results:**

In the cases with full data, common peroneal division of the sciatic nerve was affected alone or predominantly. On follow up, 72% cases showed little or partial recovery. Thirty two percent patients had residual trophic changes and causalgia at their last visit.

**Conclusion:**

The majority of cases of postinjection sciatic nerve injury have poor prognosis on conservative treatment.

## Introduction

Injection practices have been the topic of many recent studies from the developing world.[1–3] People in the South-East Asia region receive more than five injections per capita per year.[1] Data collected in cross-sectional studies in India and Pakistan have found a high frequency of indiscriminate use of injections in these regions.[2,3] More than 50% of these injections are administered in unregistered health care facilities nonformal health care systems and at home by friends and relatives for indications that may include fever, pain, infections, and injuries.[2] Common medications include antibiotics, antipyretics,[2] vitamins, and tetanus toxoid.[2] Apart from the risk of transmission of blood-borne diseases, improper injection techniques can cause peripheral nerve damage, most commonly of the radial nerve in the upper limb and the sciatic nerve in the lower limb. Studies from across the world have focused on nerve damage caused by wrong techniques of administering injections, especially in pediatric populations.[4–6] We conducted a retrospective and prospective study of the clinical and electrophysiological features of subjects with sciatic nerve injury related to intramuscular gluteal injections presenting at the Sher-i-Kashmir Institute of Medical Sciences, Srinagar, Kashmir after the injury.

## Materials and Methods

At the Sher-i-Kashmir Institute of Medical Sciences, Srinagar, Kashmir, a database is maintained of all the electrophysiological studies conducted at the laboratory. We conducted a retrospective study of this database for the period from January 2000 to December 2006. We were able to identify a total of 106 cases of post-injection sciatic nerve injury that satisfied our inclusion criteria (i.e., symptoms and/or signs in the lower limb following an intragluteal injection). The workup of the cases was done on Medelec Synergy EMG and EP system (software version 10). According to the routine at our institute, after a brief history and clinical examination, the motor nerve conduction study is done on lower limb nerves—the common peroneal and the posterior tibial. The recording is done using surface electrodes placed over the extensor digitorum brevis and the abductor hallucis longus muscles, respectively; supramaximal stimulus is used. The routine sensory nerve conduction study involves the sural and superficial peroneal nerves, with averaging of the sensory nerve action potential (SNAP) to enhance the signal-to-noise ratio. F waves are recorded over motor nerves by reversing the polarity of the stimulating electrode. The electromyographic study is conducted on the extensor digitorum brevis, tibialis anterior, medial head of gastrocnemius, and short head of biceps femoris, using a concentric needle electrode. After looking for the insertional and spontaneous activity, the motor unit action potential (MUAP) parameters of amplitude, duration, phase, and recruitment pattern are recorded (in cases where voluntary muscle contraction is intact).

The motor symptoms and signs were graded using the Medical Research Council (MRC) grading score (0-5) for knee flexion, foot dorsiflexion, and plantar flexion muscles. The grading method was a modification of that used by Eric.[7] The relative involvement of the common peroneal and posterior tibial nerves was determined by comparing the power grade in the dorsiflexors and plantar flexors and the CMAP compound muscle action potential amplitude in extensor digitorum brevis, tibialis anterior, and gastrocnemius muscles at the time of the first electrophysiological study. This was not possible in nine cases because there were only sensory abnormalities or because there was a lack of conduction abnormalities in the routine study done too early after the event. The remaining 97 patients were divided into three groups, as follows: 1) common peroneal nerve affected alone or affected more than the posterior tibial, 2) posterior tibial nerve affected alone or affected more than the common peroneal, and 3) common peroneal and posterior tibial nerves affected equally.

A total of 22 patients were followed-up in the prospective phase of the study, with clinical and electrophysiological data to assess the degree of improvement. This group of patients was divided into three outcome categories: 1) complete recovery, 2) partial recovery, and 3) no recovery. The MRC clinical grade of power 5 on last follow-up was considered to be complete recovery, even if there were some sensory symptoms. Recovery of power by >1 grade from baseline to last follow-up was taken as partial recovery; any recovery that fell short of power grade 5 was also considered as partial recovery. On the basis of increase in the amplitude of CMAP and/or SNAP, and/or EMG evidence of signs of chronic reinnervation, the group was further divided into subgroups of 1) Electrophysiological EPS recovery and 2) no electrophysiological recovery. All the patients had received splints, physiotherapy, pain medication, and vitamins during the follow-up. We used Kaplan- Meier (life table) analysis for assessing overall clinical and electrophysiological recovery in the 22 patients followed- up prospectively.

## Results

Of the 106 patients fulfilling the inclusion criteria, 84 were male and 22 female (male: female = 4:1). The ages ranged from 3 to 75 years (mean age 38 years). Thirteen cases (8.2%) were children of age 10 years or younger.

The chief complaint at presentation varied: There was postinjection weakness of the foot and leg in 59 patients (55.7%); footdrop in 36 (33.9%); and sensory symptoms of paresthesia, numbness, and pain in 20 (18.8%). The injury involved the left leg in 55.6% and the right leg in 44.4%. All patients had undergone an initial electrophysiological study at a mean of 3.8 months. (range 2 weeks to 12 months) from the onset of symptoms. The analysis of the electrophysiological features in the initial study after the injection trauma revealed two cases with decreased amplitude of SNAP in the sural nerve, without any motor conduction abnormality, and seven cases with normal motor conduction. Most of these nine cases had presented with sensory symptoms and signs only. After excluding these patients, 97 cases were available whose electrophysiological data from conduction studies and electromyography could be included for analysis. Based on motor power and CMAP amplitude, 50 patients had isolated or predominant involvement of the common peroneal nerve, 19 patients had predominant involvement of the posterior tibial nerve, and 28 patients had equal involvement of the common peroneal and posterior tibial nerves. Six patients had completely inexcitable common peroneal, posterior tibial, and sural nerves on their first study. These patients were either children or adults above the age of 55 years. CMAP was unelicitable from extensor digitorum brevis and abductor hallucis longus in 27 and 14 patients, respectively. SNAP was unelicitable from the sural and/or superficial peroneal nerves in 44 patients. Eighty-eight patients had abnormalities in the F wave, in the form of absent responses, impersistent F wave, or delayed latency compared with the normative values of the laboratory. The needle EMG revealed signs of active denervation in 25 patients and chronic reinnervation in 41 patients in the first electrophysiological study.

Twenty-two patients with complete clinical and electrophysiological follow-up were followed for 3-30 months (mean follow-up: 6.6 months) to assess the prognosis. The majority of these patients (81.8%) had sustained their nerve injury at the hands of an unqualified medical practitioner or quack unskilled medical practitioner. Analgesic injections were the offending agent in 90% of the cases. The onset of the symptoms was immediate in 90.9% of the nerve injuries and delayed in the rest. Acute or immediate onset of numbness, paresthesia, footdrop, or severe pain was the main presenting feature of nerve injury.

Fourteen patients (63.6%) showed complete or partial recovery on follow-up, whereas in 36.4% there was no clinically measurable improvement from the baseline. Six (27.2%) of the patients with complete recovery had had mainly sensory symptoms at the onset of the injury. Electrophysiological recovery was noticed in only 27% cases, and all of these achieved clinical recovery. Out of the 16 cases without recovery as per electrophysiological data, eight (50%) achieved clinical recovery as per MRC grading of power. During the follow-up seven patients (approximately 32%) developed trophic changes, mainly ulcers on the foot and causalgia.

## Discussion

Sciatic neuropathy is second commonest mononeuropathy in the lower limb after common peroneal nerve injury. The vulnerability of the nerve to damage is attributed to its long anatomic course from the lumbosacral plexus, through the sciatic notch, up to its bifurcation just above the popliteal fossa. Damage to the sciatic nerve by injection has been reported from several parts of the world, including South-East Asia; these injuries are related mainly to a faulty injection technique and the lack of trained manpower capable of administering parenteral drugs in the field in the developing countries.[1–3] Sciatic nerve injury following intragluteal injection constituted only 2.7% of the cases in one series of sciatic neuropathy from the University of California.[7] However, a review of literature ranked it the second most common cause of sciatic nerve injury after hip arthroplasty.[8] Injection injury accounted for 50% of the sciatic nerve injuries in one large series reported from the USA.[5]

The literature from India on this subject is based on experience with the pediatric population.[4,6,9] In the present study, the bulk of the patients (91%) were above 10 years of age and the offending agent was most commonly an analgesic, e.g., diclofenac sodium, which is administered frequently for all kinds of pain. Antibiotic injections are reported to be the main culprits in infants and children. Eighty-one percent of the cases in the present series had been administered the injection by unqualified medical practitioner or quack an unskilled medical practitioner, a fact that has been previously highlighted by another study from Ludhiana in India.[10] The frequent and unwarranted use of injections, faulty technique, and the fact that there is not much gluteal muscle mass in children and emaciated subjects are all responsible. Thus, sciatic nerve injury is a common cause of avoidable disability worldwide.[9,11]

The loss of SNAP of the sural and superficial peroneal nerves and the greater vulnerability of the common peroneal nerve to injury, that was apparent in the present series, has been noticed previously also in cases of injection injury as well as in injuries caused by other mechanisms.[7,10,12] However, the present series highlights the good prognosis in patients who have sensory nerve conduction abnormalities alone in the first electrophysiological study after the injury. The electrophysiological finding of completely inexcitable common peroneal, posterior tibial, and sural nerves was seen in children and in those above the age of 55 years in this series, reflecting the greater degree of nerve damage in these age-groups. Such cases showed poor clinical recovery. The poor outcome in the form of no recovery or partial recovery in 72% of the cases in our prospectively followed population is consistent with the results of two large Indian series in pediatric and adult populations, which reported that 64% and 82% of cases, respectively, were left with permanent residual deficits on conservative management of the sciatic nerve injury following intragluteal injection.[4,10] The present case series also highlights the lack of significant clinical recovery beyond 15 months of follow-up; clinical recovery can occur in some cases despite there being no objective recovery in the electrophysiological parameters. The poor outcome in the present study as well as in other Indian studies can be attributed to: 1) nonfeasibility of immediate saline irrigation (a treatment measure proposed by some authors)[14,15] because the majority of such injuries in India are sustained in the rural setup and 2) the failure to refer such cases for specialized procedures like neurolysis and tendon transfer techniques which can yield good results.[16,17] In view of these realities, we agree with the suggestion of Ahuja *et al.*[4] that to prevent this avoidable nerve injury the practice of intramuscular injection in the gluteal region, especially in the high-risk cases like children and the elderly, should be abandoned. Also, as recommended by Gilles and French,[11] in case of persistent neurodeficit on conservative care, the patient should undergo neurolysis. The underlying histopathological features of nerve fibrosis and tuberculoma formation[11,13] in children after injection injury to sciatic nerve can also be dealt with by neurolysis[11] to prevent long-term effects like limb shortening and clubfoot deformity in children. The presence of extensor digitorum brevis compound muscle action potential within 6 months and early recovery of function have been found to be good prognostic indicators for recovery.[7,18] However, the discordance between the electrophysiological recovery and the clinical recovery and the high incidence (32%) of trophic foot ulcers and causalgia on prolonged follow-up in the present study, together with the issue of the difficulty in determining reinnervation as reported by Midha *et al*.,[19] leaves the question of the ideal timing of the surgical intervention unresolved.
